# N-3 PUFA Deficiency Affects the Ultrastructural Organization and Density of White Matter Microglia in the Developing Brain of Male Mice

**DOI:** 10.3389/fncel.2022.802411

**Published:** 2022-02-10

**Authors:** Fanny Decoeur, Katherine Picard, Marie-Kim St-Pierre, Andrew D. Greenhalgh, Jean-Christophe Delpech, Alexandra Sere, Sophie Layé, Marie-Eve Tremblay, Agnès Nadjar

**Affiliations:** ^1^INRAE, Bordeaux INP, NutriNeuro, Université de Bordeaux, Bordeaux, France; ^2^Axe Neurosciences, Centre de Recherche du CHU de Québec–Université Laval, Québec, QC, Canada; ^3^Division of Medical Sciences, University of Victoria, Victoria, BC, Canada; ^4^Département de Médecine Moléculaire, Université Laval, Québec, QC, Canada; ^5^Department of Neurology and Neurosurgery, McGill University, Montreal, QC, Canada; ^6^Department of Biochemistry and Molecular Biology, The University of British Columbia, Vancouver, BC, Canada; ^7^Neurocentre Magendie, U1215, INSERM-Université de Bordeaux, Bordeaux, France; ^8^Institut Universitaire de France (IUF), Paris, France

**Keywords:** n-3 PUFA, microglia, myelin, neurodevelopment, electron microscopy

## Abstract

Over the last century, westernization of dietary habits has led to a dramatic reduction in dietary intake of n-3 polyunsaturated fatty acids (n-3 PUFAs). In particular, low maternal intake of n-3 PUFAs throughout gestation and lactation causes defects in brain myelination. Microglia are recognized for their critical contribution to neurodevelopmental processes, such as myelination. These cells invade the white matter in the first weeks of the post-natal period, where they participate in oligodendrocyte maturation and myelin production. Therefore, we investigated whether an alteration of white matter microglia accompanies the myelination deficits observed in the brain of n-3 PUFA-deficient animals. Macroscopic imaging analysis shows that maternal n-3 PUFA deficiency decreases the density of white matter microglia around post-natal day 10. Microscopic electron microscopy analyses also revealed alterations of microglial ultrastructure, a decrease in the number of contacts between microglia and myelin sheet, and a decreased amount of myelin debris in their cell body. White matter microglia further displayed increased mitochondrial abundance and network area under perinatal n-3 PUFA deficiency. Overall, our data suggest that maternal n-3 PUFA deficiency alters the structure and function of microglial cells located in the white matter of pups early in life, and this could be the key to understand myelination deficits during neurodevelopment.

## HIGHLIGHTS

- Low n-3 PUFA intake reduces white matter microglia density at key stage during development.

- Low n-3 PUFA intake reduces the interactions of white matter microglia with myelin.

- Low n-3 PUFA intake increases the abundance of mitochondria within white matter microglia.

## Introduction

The central nervous system (CNS) is mainly composed of lipids. Among these, the most abundant lipid species in the brain parenchyma are polyunsaturated fatty acids (PUFAs), more specifically arachidonic acid (AA, 20:4 n-6) and docosahexaenoic acid (DHA, 22:6 n-3) (Sastry, 1985; Rapoport et al., 2007; Betsholtz, 2015). AA and DHA can be biosynthesized from their precursors, respectively, linoleic acid (LA, 18:2 n-6) and α-linolenic acid (ALA, 18:3 n-3), or directly provided by the diet (Lands et al., 1990). Brain accumulation of PUFAs, especially DHA, during critical periods is indispensable for CNS development and functional maturation in humans (Clandinin et al., 1980; Lauritzen et al., 2005; Innis, 2007; McNamara et al., 2017). The global reduction in dietary n-3 PUFA intake, witnessed over the last century, has led to a decrease in the brain concentration of DHA and AA (Lands et al., 1990; Innis, 2007; Koletzko et al., 2008; Bradbury, 2011; Stark et al., 2016). Low intake of n-3 PUFAs has raised concerns about its potential adverse effects on the neurological development of human infants (Koletzko et al., 2008; McNamara et al., 2015, 2017; Madore et al., 2016) resulting in an increased incidence of neurodevelopmental disorders (McNamara et al., 2015; Martinat et al., 2021).

We and others have shown that n-3 PUFA deficiency during brain development impairs microglial physiology, microglia-mediated synaptic refinement, axon myelination in the white matter, neuritogenesis and neurotransmission, leading to a wide range of neurobehavioral abnormalities (Pifferi et al., 2005; Chalon, 2006; Coti Bertrand et al., 2006; Kawakita et al., 2006; Beltz et al., 2007; Cao et al., 2009; Yavin et al., 2009; Lafourcade et al., 2011; de Velasco et al., 2012; Madore et al., 2014, 2020; Leyrolle et al., 2021a). Despite these observations, the mechanisms by which n-3 PUFA deficiency affects proper CNS development, specifically myelination, require further studies.

A series of recent publications revealed a crucial microglial role in regulating myelination during neurodevelopment (Shigemoto-Mogami et al., 2014; Hagemeyer et al., 2017; Wlodarczyk et al., 2017; Hughes and Appel, 2019; Li et al., 2019; Sierra et al., 2019; Stratoulias et al., 2019; Benmamar-Badel et al., 2020; Nemes-Baran et al., 2020; Tanabe and Yamashita, 2020; Traiffort et al., 2020; Fujita and Yamashita, 2021; Kalafatakis and Karagogeos, 2021). Microglia invade the white matter early after birth, during the first post-natal days in mice (Stratoulias et al., 2019; McNamara and Miron, 2020). Depleting the brain of microglia at this developmental period impairs the proper formation of oligodendrocyte progenitors (OPCs) and reduces post-natal myelination (Hagemeyer et al., 2017). Microglia sustain myelination by releasing growth factors such as the insulin-like growth factor 1 (IGF-1) and galectin-3, which promote the survival, differentiation and maturation of OPCs (Pasquini et al., 2011; Miron et al., 2013; Pang et al., 2013; Wlodarczyk et al., 2017). They also contribute to white matter formation by phagocyting fragments of myelin sheaths, or by engulfing supernumerary OPCs, two essential processes for normal brain myelination (Liu et al., 2013; Hughes and Appel, 2019; Nemes-Baran et al., 2020).

Based on previous results, we hypothesized that the defects in myelination observed in the white matter of n-3 PUFA deficient pups could result from an impairment of white matter microglia. To test this hypothesis, we analyzed the impact of n-3 PUFA deficiency on microglia in the developing white matter. We first quantified microglial density in the main white-matter tract within the brain, the *corpus callosum*, of the offspring across different post-natal ages (P0, P5, P10, and P15). We found a significant reduction in the number of white matter microglia in the brain of n-3 PUFA deficient mice at P10. We further characterized the ultrastructure of the *corpus callosum* microglial population at P10, using qualitative and quantitative transmission electron microscopy analyses. We observed significantly fewer contacts between microglia and myelinated axons, as well as fewer myelin fragments within microglial cells under n-3 PUFA deficiency. These findings were correlated with an increase in the abundance of mitochondria in microglial cells, but not of the ultrastructure of this organelle. In conclusion, our results suggest that maternal n-3 PUFA deficiency alters the structure of microglial cells located in the developing *corpus callosum*, as well as their interactions with the local environment.

## Experimental Procedure

### Animals

All experiments were approved and performed under the guidelines of the local and national ethics committee for care and use of animals (Direction Départementale de la Protection des Animaux, approval ID: A33-063-920, #15517) and carried out according to the Quality Reference System of INRAE and the Directive 2010/63/EU. Every effort was made to minimize the number of animals used (Janvier Labs, France) and their suffering. CD1 mice were maintained in standard cages in mixed litter with a 12 h light-dark cycle, temperature and humidity controlled, and food and water provided *ad libitum*. To reduce the use of animals, the same brains were studied for light and electron microscopy, *n* = 4–6/group. Litter size at birth is around 12 pups per dam. CD1 juvenile (P0, P5, P10, P15) males were used for the experiments as our previous results, where we observed myelination alterations, were also obtained in male mice (Leyrolle et al., 2021b). Moreover, the literature on microglia/myelin interactions focuses on males (Hagemeyer et al., 2017; Hughes and Appel, 2019; Li et al., 2019).

### Diet

During gestation and lactation, CD1 dams (obtained from Janvier Labs at 2 months of age) were fed with isocaloric diets (from INRAE Jouy en Josas) containing 5% fat with low (n-3 PUFA sufficient group) or high (n-3 PUFA deficient group) n-6/n-3 precursor ratio, respectively, α-linolenic acid (ALA, 18:3 n-3) and linoleic acid (LA, 18:2 n-6) (detailed composition is reported in Tables 1, 2), according to the international nutritional recommendations and as previously published (Delpech et al., 2015; Labrousse et al., 2018; Decoeur et al., 2020; Madore et al., 2020; Leyrolle et al., 2021a,b). We could not observe any effect of the diet on maternal behavior (e.g., active nursing, pups licking/sniffing, nesting) or litter size (unpublished observations. We previously reported that decreasing the amount of n-3 PUFA precursors in the maternal diet led to a significant increase in the n-6 PUFA/n-3 PUFA ratio in the pups’ brain structures, white matter and microglial cells (Joffre et al., 2016; Madore et al., 2020; Leyrolle et al., 2021b).

**TABLE 1:** Fatty acid composition of the dietary lipids (% by weight of total fatty acids).

Diets	Sufficient	Deficient
16:00	22.6	6.2
18:00	3.3	4.4
Other saturated FAs	1.8	1.6
Total saturated FAs	27.7	12.2
16:1n-7	0.2	0.1
18:1n-9	57.9	26.0
18:1n-7	1.5	0.9
Other monounsaturated FAs	0.4	0.2
Total monounsaturated FAs	60.0	27.2
18:2n-6 (LA)	10.6	60.5
20:4n-6 (AA)	ND	ND
Total n-6 PUFAs	10.7	60.5
18:3n-3 (ALA)	1.6	0.1
18:4n-3	ND	ND
20:5n-3	ND	ND
22:5n-3	ND	ND
22:6n-3 (DHA)	ND	ND
Total n-3 PUFAs	1.6	0.1
total PUFAs	12.3	60.6
n-6/n-3	6.7	> 500

*FAs, fatty acids; LA, linoleic acid; AA, arachidonic acid; PUFAs, polyunsaturated fatty acids; ALA, α-linolenic acid; ND, not detected (under the limit for the detection by gas).*

**TABLE 2:** Composition of the diets (g/kg diet).

Ingredient	Amount
Casein	180
Cornstarch	460
Sucrose	230
Cellulose	20
Fat ^1^	50
Mineral mix ^2^	50
Vitamin mix ^3^	10

*^1^for detailed composition, see Table 1.*

*^2^composition (g/kg): sucrose, 110.7; CaCO3, 240; K2HPO4, 215; CaHPO4, 215; MgSO4,7H2O, 100; NaCl, 60; MgO, 40; FeSO4,7H2O, 8; ZnSO4,7H2O, 7; MnSO4,H2O, 2; CuSO4,5H2O, 1; Na2SiO7,3H2O, 0.5; AlK(SO4)2,12H2O, 0.2; K2CrO4, 0.15; NaF, 0.1; NiSO4,6H2O, 0.1; H2BO3, O.1; CoSO4,7H2O, 0.05; KIO3, 0.04; (NH4)6Mo7O24,4H2O, 0.02; LiCl, 0.015; Na2SeO3, 0.015; NH4VO3, 0.01.*

*^3^composition (g/kg): sucrose, 549.45; retinyl acetate, 1; cholecalciferol, 0.25; DL-a-tocopheryl acetate, 20; phylloquinone, 0.1; thiamin HCl, 1; riboflavin, 1; nicotinic acid, 5; calcium pantothenate, 2.5; pyridoxine HCl, 1; biotin, 1; folic acid, 0.2; cyanobalamin, 2.5; choline HCl, 200; DL-methionin, 200; p-aminobenzoic acid, 5; inositol, 10.*

### Perfusion and Cutting for Light and Electron Microscopy

Mouse pups were anesthetized with 10% lurocaine and 5% exagon. Blood was intracardially flushed with ice-cold phosphate-buffered saline solution (pH 7.4, 50 mM) (PBS) followed by a fixation with a 0.2% glutaraldehyde in 4% paraformaldehyde (PFA, Sigma) solution. Brains were extracted and post-fixed in 4% PFA at 4°C for 3 h.

Total of 50-μm coronal brain sections (Bregma −0.18 to −2.55) were generated using a vibratome (Leica VT1000 S) and stored at −20°C in a cryoprotectant solution (30% glycerol and 30% ethylene glycol in PBS) prior to the experiments.

### Iba1 (Ionized Calcium-Binding Adapter Molecule 1) Immunostaining and Analyses by Light Microscopy

Briefly, free-floating sections containing the region of interest (*corpus callosum*) were washed and quenched for 30 min in 0.3% H_2_O_2_ in H_2_O. Sections were incubated in a blocking buffer (BB) solution (3% bovine serum albumin, 0.3% Triton X-100 in PBS) at room temperature (RT) for 1 h. Then, samples were immunostained using a rabbit polyclonal anti-Iba1 primary antibody (1/1,000, Wako cat#019-19741, Neuss, Germany) diluted in BB at 4°C overnight. The following day, the sections were washed and incubated with a biotinylated goat anti-rabbit secondary antibody (1/2,000, Invitrogen, Saint Aubin, France) for 2 h at RT, followed by a 1 h incubation in a peroxidase conjugated avidin/biotin complex solution (Vectastain ABC kit Biovalley) at RT. The staining was revealed using 0.6% diaminobenzidine (DAB) with a nickel-enhanced glucose oxidase method (Shu et al., 1988). The sections were washed, dehydrated in increasing concentrations of ethanol, mounted on slides and coverslipped using Depex mounting medium (BDH, 361254D).

Sections (*n* = 4–6/group) containing the *corpus callosum* (body; −0.18 to −2.55 to Bregma) were imaged at 20X using a Nanozoomer slide scanner (Hamamatsu Photonics), extracted with the NDP.view 2 freeware (Hamamatsu Photonics) and analyzed with ImageJ software. The *corpus callosum* area traced was calculated and the density of Iba1 + cells was determined per mm^2^. The analysis was conducted blind to the experimental conditions. Brain sections were obtained from 6 P0 litters (for a total of 4 n-3 sufficient = mice and 5 n-3 deficient mice), 5 P5 litters (n-3 sufficient = 4 mice, n-3 deficient = 4 mice), 5 P10 litters (n-3 sufficient = 4 mice, n-3 deficient = 5 mice), 4 P15 litters (n-3 sufficient = 6 mice, n-3 deficient = 5 mice).

### Iba1 Immunostaining and Ultrastructural Analysis by Electron Microscopy

Sections containing the *corpus callosum* were washed with PBS then quenched in 0.3% H_2_O_2_ in PBS for 5 min and permeabilized in 0.1% NaBH_4_ in PBS for 30 min. Samples were incubated 1 h at RT in BB (10% fetal bovine serum, 3% bovine serum albumin, 0.01% Triton X-100, diluted in PBS) before immunostaining with rabbit anti-Iba1 primary antibody (1/1,000 in BB; same antibody as above) overnight at 4°C. Samples were washed in TBS and incubated with biotinylated goat anti-rabbit secondary antibody (1/300 in TBS; cat# 111-066-046, Jackson ImmunoResearch, West Grove, PA, United States) 2 h at RT, followed by a peroxidase conjugated avidin/biotin complex solution (Vectastain ABC kit Biovalley) 1 h at RT. Immunostaining was revealed in 0.05% DAB containing 0.015% H_2_O_2_. Sections were washed and incubated in 3% potassium ferrocyanide in milliQ H_2_O combined (1:1) with 4% (aq) osmium tetroxide (cat# 19170, Electron Microscopy Sciences, Hatfield, PA, United States) for 1 h, then incubated in heated 1% thiocarbohydrazide (in PBS; cat# 2231-57-4, Electron Microscopy Sciences) diluted in milliQ H_2_O for 20 min, and incubated in 2% osmium tetroxide (diluted in H_2_O) for 30 min. Sections were dehydrated in increasing concentrations of ethanol, followed by propylene oxide. Tissues were infiltrated with Durcupan ACM resin (cat# 44611-44614, MilliporeSigma) overnight at RT and flat-embedded between fluoropolymer sheets at 55°C for 5 days. The *corpus callosum* (body) was excised from the sections and re-embedded on resin blocks. Ultra-thin sections were cut at 70 nm using a Leica ARTOS 3D Ultramicrotome. Sections were collected and placed on copper square mesh grids (cat#G150-Cu Electron Microscopy Sciences). Pictures were acquired at a magnification of X10000 under a JOEL JEM-1400 transmission electron microscope operated at 80 kV using a Gatan SC-1000 digital camera (11 Megapixel CCD camera).

Images of 8 to 12 microglial cell bodies per animal (*n* = 63–65 microglia/diet, *N* = 6 mice/group) were analyzed by an experimenter blinded to the experimental conditions using ImageJ. Microglial cell bodies were recognized by their Iba1-immunopositive (+) cytoplasm (dark staining, dense to electrons), nuclear hetero- and euchromatin pattern characteristic of these cells, as well as long and narrow cisternae of endoplasmic reticulum (ER), among other distinctive features (Tremblay et al., 2010; Nahirney and Tremblay, 2021). Microglial cells are phagocytes and therefore it is common to find inclusions in their cytoplasm. Microglia organelles are distributed everywhere in the cytoplasm, unlike oligodendrocytes, whose organelles tend to be arranged in one place. Ultrastructural analyses of microglial cell bodies were performed, determining their: (1) shape: area, perimeter, circularity (area-to-perimeter ratio), aspect ratio (ratio of the objects height to width), roundness (is defined by the ratio of the area of an object to the area of a circle with the same convex perimeter; excludes local irregularities) and solidity (density of an object, area-to-convex area ratio); (2) organelle number: ER, Golgi apparatus, lysosomes, empty phagosomes with a diameter larger than 100 nm, phagosomes containing parenchymal elements including myelin and mitochondria; (3) organelles anomalies: dilation of the ER and Golgi apparatus, alteration or elongation of mitochondria; (4) parenchymal interactions: contacts with axon terminals, dendritic spines, myelinated axons, astrocytic and neuronal cell bodies. Dendritic spines are recognized by their postsynaptic density and axon terminals by their synaptic vesicles. To analyze the contacts with excitatory synapses (combination of both a pre- and a post-synapse), we considered the elements directly juxtaposing the microglial cell bodies. Previously described identification criteria were used for the ultrastructural analysis (Bisht et al., 2016; Chavez-Valdez et al., 2016; El Hajj et al., 2019; St-Pierre et al., 2019; Lecours et al., 2020; Savage et al., 2020; Bordeleau et al., 2021; Nahirney and Tremblay, 2021). For instance, mitochondria were recognized by their double-membrane, their oval shape and electron-dense appearance with the presence of cristae. We analyzed the percent area occupied by the overall mitochondrial network (cytoplasmic fraction containing mitochondria). Altered mitochondria were identified by their swollen appearance, broken cristae, as well as holy-shape or elongated form (more than 1,000 nm long). The ER cisternae were distinguished by their long and narrow shape, while the Golgi apparatus is shorter, circular and appeared as a stack of elongated circular profiles on top of each other. Phagosomes were identified by their defined electron-dense membrane containing parenchymal elements including myelin. Myelinated axons were characterized by their electron-lucent cytoplasm surrounded by electron-dense sheaths (Lampron et al., 2015; Nahirney and Tremblay, 2021). Oligodendrocyte cell bodies were recognized by their electron-dense cytoplasm with patchy heterochromatin pattern and organelles were generally distributed to one side of the cell. Neuronal cell bodies were identified by a granular appearance of their electron-lucent (light gray) nucleo- and cytoplasm. These cells were circular and much larger than other brain cells. Astrocyte cell bodies were recognized by their electron-lucent cyto- and nucleoplasm with angular shaped protrusions from the plasma membrane and the presence of intermediary filaments (Bisht et al., 2016; Chavez-Valdez et al., 2016; El Hajj et al., 2019; St-Pierre et al., 2019; Lecours et al., 2020; Savage et al., 2020; Bordeleau et al., 2021; Nahirney and Tremblay, 2021).

### Statistical Analyses

All statistical analyses were performed by using Prism 9 (GraphPad software). The results are expressed as mean ± standard error of the mean (SEM), except Table 3 which presents medians. Statistical significance was set to *p* < 0.05. Normality and homoscedasticity were verified using the Shapiro–Wilk test and Brown-Forsythe test, respectively. Non-parametric unpaired two-tailed Student’s *t*-tests were applied for all analyses that did not pass normality and/or homoscedasticity. None of the microglial ultrastructure parameters presented in Table 3 pass normality using Shapiro–Wilk test. They were analyzed by non-parametric two-tailed Student’s unpaired *t*-tests. The analysis of microglial properties was usually conducted using *n* = cell considering the biological unit as the microglia, and not the animal. This is similar to what is done with other cell types including microglia and their compartments by our group and others (Hellwig et al., 2016; Milior et al., 2016; Lecours et al., 2020).

**TABLE 3:** Quantification of ultrastructural parameters in P10 mice.

	n-3 sufficient	n-3 deficient	*p*-value
Nucleus area (μm^2^)	14.31	16.62	0.16
Cytoplasmic area (μm^2^)	7.48	8.93	0.02
Nucleus perimeter (μm)	17.22	18.88	0.16
Cytoplasmic perimeter (μm)	26.36	29.47	0.27
Circularity	0.66	0.63	0.1
Aspect Ratio	2.11	2.07	0.63
Roundness	0.47	0.48	0.67
Solidity	0.95	0.95	0.71
Phagosomes per cell (*n*)	2	1	0.27
ER dilation (% of cells)	0	0	0.4
Golgi dilation (% of cells)	0	0	0.24
Myelin debris per cell (*n*)	0	0	0.01
Mitochondria per cell (*n*)	3	5	0.001
Elongated mitochondria per cell (*n*)	0	0	0.80
Altered mitochondria per cell (*n*)	0	0	0.16
Percent cytoplasm occupied by mitochondria (% of area)	3.62	6.20	0.002
Contact with myelinated axon per cell (*n*)	0	0	0.02
Contact with oligodendrocyte per cell (*n*)	0	0	0.55
Contact with astrocyte per cell (*n*)	0	0	>0.99
Contact with neuron per cell (*n*)	0	0	>0.99
Contact with pre-synapse per cell (*n*)	1	0	0.18
Contact with post-synapse per cell (*n*)	0	0	0.07
Contact with pre- and post-synapse per cell (*n*)	0	0	0.60

*a.u., arbitrary unit; statistical significance was set to p < 0.05.*

*The results are expressed as median ± SEM.*

*Non-parametric unpaired two-tailed Student’s t-tests were applied for all analyses.*

## Results

### Dietary n-3 PUFA Deficiency Affects Microglial Density in the Developing *Corpus callosum*

**FIGURE 1:**
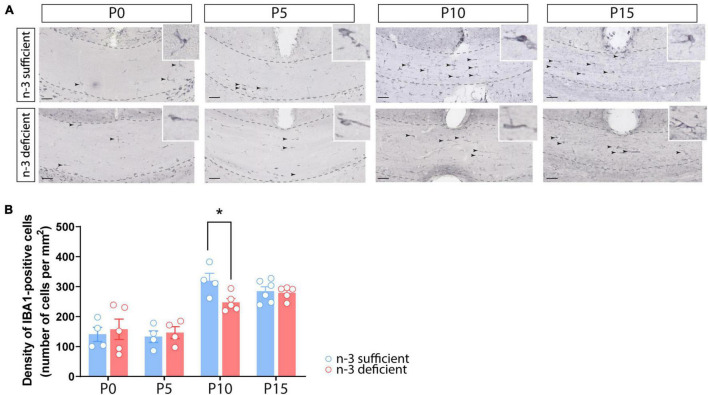
Dietary n-3 PUFA deficiency decreases microglial density in the developing *corpus callosum*. **(A)** Representative images of Iba1-positive cells (arrow) at different post-natal ages (P0, P5, P10, and P15) in the *corpus callosum* of n-3 sufficient and n-3 deficient mice (scale bar = 50 μm; dotted lines delineate the *corpus callosum*). **(B)** Result of the quantification of Iba1-positive cell density, at different post-natal ages (P0, P5, P10, and P15) in the *corpus callosum* of n-3 sufficient and n-3 deficient mice. All data are presented as mean ± SEM. Blue bars/circles = n-3 sufficient mice, red bars/circles = n-3 deficient mice. *n* = 4–6 animals/group. *p < 0.05.

We first quantified the number of white matter microglia using Iba1 immunostaining in the *corpus callosum* of n-3 sufficient and n-3 deficient mice, at post-natal ages P0, P5, P10, and P15, i.e., when microglia are expected to invade the white matter (Hagemeyer et al., 2017). Iba1 + microglial cell density was similar between both dietary groups at P0, P5, and P15. However, n-3 deficient mice displayed less Iba1 + cells at P10, compared to control animals (Figures 1A,B) (P10 n-3 sufficient *vs* n-3 deficient: Unpaired *t*-test: *t* = 2,701 df = 7, *p = 0.0306). While we cannot firmly conclude on the total number of microglia, this shows that n-3 PUFA deficiency affects microglial cell density in the white matter, at a key stage of neurodevelopment, and highlights temporal abnormalities in the post-natal colonization of the *corpus callosum* by microglia.

### N-3 PUFA Deficiency Reduces Microglial Interactions With Myelin in the Developing *Corpus callosum*

**FIGURE 2:**
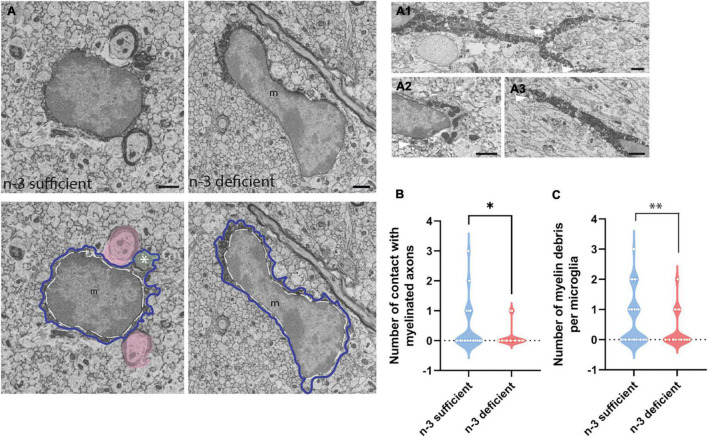
N-3 PUFA deficiency reduces the number of contacts between microglia and myelin axon and decreases the presence of myelin fragments in microglia. **(A)** Representative images of microglial ultrastructure in the *corpus callosum* of P10 mice (left panel: n-3 sufficient, right panel: n-3 deficient; upper panels: original images, lower panels: with false colors), showing interactions between microglial cell bodies and myelinated axons (in pink), as well as myelin debris (green, see asterisk) in the cytoplasm of the cell (magnification: 10,000×) (scale bar = 1 μm). More examples of microglia engulfing myelin debris (see asterisk and arrowhead) in A1–A3; Scale bar = 1 μm. Microglia are annotated with “m,” the contour of their nucleus is delineated with a white dotted line and the cytoplasm by a purple line. **(B)** Quantification of the number of myelinated axons in contact with microglia in both dietary groups. **(C)** Quantification of number of myelin debris in microglial cells, in both dietary groups. All data are presented as mean ± SEM. *n* = 63–65 microglia/group, *N* = 6 mice/group.

To study at nanoscale resolution whether and how white matter microglia of the *corpus callosum* interact with their microenvironment, we conducted transmission electron microscopy imaging and analyses on brain slices from P10 pups (based on our previous observation of microglial density differences). We quantified the number of contacts between microglia and myelinated axons, axon terminals, dendritic spines, oligodendrocytes, astrocytes, and neurons (Table 3). While most of the parameters studied were not significantly different between n-3 sufficient and n-3 deficient mice (Table 3), we observed a significant decrease in the number of contacts per cell between microglia and myelinated axons (Figures 2A,B and Table 3) (n-3 sufficient *vs* n-3 deficient: Mann–Whitney test *p = 0.0227). Moreover, 29% of microglia contacted myelinated axons in the n-3 sufficient condition *vs* 14% in n-3 deficient mice (data not shown). We also assessed the number of myelin fragments per microglial cell body and found significantly less myelin debris in microglia from n-3 deficient mice (Figures 2A,C and Table 3) (n-3 sufficient *vs* n-3 deficient: Mann–Whitney test ^**^p = 0.009). These data indicate that not only are there fewer microglia, but low n-3 PUFA intake affected the interaction of microglia with myelin in the *corpus callosum*.

### N-3 PUFA Deficiency Increases Microglial Abundance of Mitochondria in the Developing *Corpus callosum*

**FIGURE 3:**
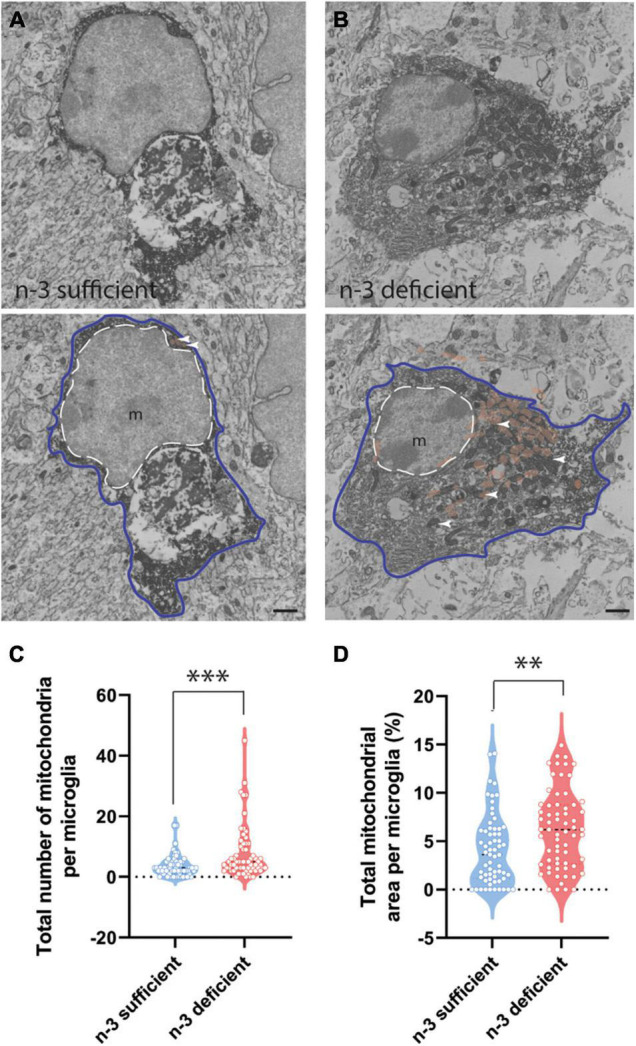
N-3 PUFA deficiency increases mitochondrial abundance and area in microglia at P10. **(A,B)** Representative images of microglial ultrastructure in the *corpus callosum* of P10 mice, showing the abundance of mitochondria (upper panels: original images, lower panels: with false colors: e.g., mitochondria in orange + white arrowheads) (magnification: 10,000×) (scale bar = 1 μm) in n-3 sufficient mice **(A)** and n-3 deficient mice **(B)**. Microglia are annotated with “m,” the contour of their nucleus is delineated with a white dotted line and the cytoplasm by a purple line. Mitochondria are highlighted in false color (light brown). **(C)** Quantification of number of mitochondria in microglia cells from both dietary groups. **(D)** The percent of cytoplasm containing mitochondria in both dietary groups. All data are presented as mean ± SEM. *n* = 63–65 microglia/group, *N* = 6 animals/group. **p < 0.01, ***p < 0.001.

To further characterize the ultrastructure of white matter microglia, we quantified their cellular content in the *corpus callosum* of P10 pups, including mitochondria, an intracellular organelle whose primary function is to provide the energy that microglia need to survive. It has been recently shown that microglia control their states and functional properties by adapting intracellular metabolic pathways to their energy requirements (Nadjar, 2018; Borst et al., 2019; Paolicelli and Angiari, 2019; Lauro and Limatola, 2020). Microglial cell bodies exposed to n-3 PUFA deficient diet displayed a significant increase in the number of mitochondria (Figures 3A–C and Table 3) (n-3 sufficient *vs* n-3 deficient: mitochondria, Mann–Whitney test ^***^p = 0.0008). Since we observed an increase of microglial cytoplasmic area (Table 3) (n-3 sufficient *vs* n-3 deficient: Mann–Whitney test *p = 0.0239), and to avoid any bias, we then analyzed the percent area occupied by the overall mitochondrial network (cytoplasmic fraction containing mitochondria). N-3 PUFA deficient diet caused a significant increase in the mitochondrial network area (Figures 3A–D and Table 3) (n-3 sufficient *vs* n-3 deficient: percent cytoplasm containing mitochondria, Mann–Whitney test ^**^p = 0.0023). This was not accompanied by an increase in markers of cellular stress in the mitochondria (Altered mitochondria can be identified by their swollen appearance, broken cristae, showing a holy-shape and elongated form, more than 1,000 nm long) (Table 3). These observations suggest an alteration of microglial energy metabolism under n-3 PUFA deficiency, in the *corpus callosum* of P10 pups. We also studied other microglial organelles including the ER, phagosome and Golgi apparatus. The morphology of these organelles was not significantly different between n-3 sufficient and n-3 deficient mice (Table 3).

## Discussion

Overall, our results showed that maternal n-3 PUFA deficiency affects white matter microglia in the early post-natal offspring. We observed a decrease in Iba1 + microglial density in the *corpus callosum* at P10, together with less microglial contacts with myelin, less internalized myelin fragments and increased abundance and area of mitochondria. We acknowledge the need to study females in a follow-up study, considering the described sexual dimorphism of microglia and myelination processes (Cerghet et al., 2009; Han et al., 2021).

We previously observed that microglia from the gray matter are affected by n-3 PUFA deficiency at P21 (Madore et al., 2014, 2020). More specifically, we showed that microglia located in the hippocampus are profoundly altered in their lipid composition (less n-3 PUFAs and more n-6 PUFAs in their membranes), in their physiology (more prone to react to inflammatory stimuli, more phagocytic) and in their motility (processes are less motile), when exposed to low n-3 PUFAs (Madore et al., 2014, 2020). We also revealed that these modifications are causal to the neuronal deficits observed in n-3 deficient mice, including impairment of spatial working memory (Madore et al., 2020). In the present study, we observed that microglia located in the white matter are also compromised in the offspring at P10, when maternal n-3 PUFA dietary intake is low, affecting the ultrastructural organization and density of the white matter microglial population among the *corpus callosum*. We chose the latter based on the previous studies on myelin/microglia interactions during development (Hagemeyer et al., 2017; Kaur et al., 2017; Lee et al., 2019; Nemes-Baran et al., 2020; Traiffort et al., 2020). This is also in this area that we observed an alteration of myelination in mice deficient in n-3 PUFA in a former study (Leyrolle et al., 2021b). We focused on one part of the *corpus callosum*, the body that unites the motor, somatosensory and parietal regions (Wahl et al., 2007; Park et al., 2008; Putnam et al., 2010; Ku and Torii, 2020). More experiments are needed to fully unravel to what extent n-3 PUFA deficiency affects microglial morphology, lipid composition and functions in the *corpus callosum* and how this would relate to behavior abnormalities later in life.

Our data reveal that n-3 deficient mice did not display the peak in microglial density classically observed at P10. Indeed, according to the literature, microglia invade the *corpus callosum* of mice, a phenomenon called the “fountain of microglia” (Del Rio-Hortega and Penfield, 1932; Imamoto and Leblond, 1978; Ling, 1979), with a peak around that age, to promote adequate induction of oligodendrocytes (Hagemeyer et al., 2017; Wlodarczyk et al., 2017). A reversible depletion of microglia during this time frame reduces the total number of mature oligodendrocytes at P20, even if the total number of microglia is back to normal at that age (Hagemeyer et al., 2017). This suggests that the peak of microglial invasion occurring at P10 is essential for proper myelination of the brain. In line with this assumption, Kalafatakis and Karagogeos, 2021 have shown that the time course of microglial migration to the white matter correlates with the onset of OPCs generation in this structure. Interestingly, we previously demonstrated that maternal n-3 PUFA deficiency leads to hypomyelination of the corpus callosum at P21 and in the adult offspring, and to a significant decrease in the number of mature oligodendrocytes in the *corpus callosum* of juveniles, associated with long-term deficits in brain structures synchronization (Leyrolle et al., 2021b). Hence, while the total number of microglia was similar between both dietary groups at P15, the absence of a peak at P10 in n-3 deficient mice may have significant functional impacts. More studies are needed to fully test this hypothesis.

During development, white matter microglia phagocytose excess myelin sheaths in an activity-dependent manner, a key process for optimal myelination (Hughes and Appel, 2019; Zareba and Peri, 2021). Here, we observed that microglia from n-3 deficient animals contained less myelin fragments and were interacting less with myelinated axons. This finding suggests that myelin turnover is less efficient in these animals, which might explain our previous result that n-3 PUFA deficiency decreases myelin thickness in the *corpus callosum* of mouse pups, resulting in a long-term decrease of brain functional connectivity (Leyrolle et al., 2021b). In line with this assumption, we previously showed that treating mouse pups with clemastine, a first-generation histamine antagonist and pro-myelinating agent, could restore OPCs density in the *corpus callosum* of n-3 deficient mice (Leyrolle et al., 2021b). The mechanisms of action of clemastine are still a matter of debate. Indeed, clemastine could promote OPCs maturation and proliferation, not only *via* a direct action, but also *via* signaling through M1 muscarinic receptors expressed by microglia (Cree et al., 2018; Lloyd and Miron, 2019). In turn, microglia could promote OPCs maturation and myelination (Shigemoto-Mogami et al., 2014; Su et al., 2018; Xie et al., 2020). More studies are needed to understand whether the reduced uptake of myelin debris by microglia that we found in n-3 deficient mice is responsible for the deficits in myelin previously observed in these animals (Leyrolle et al., 2021b).

Polyunsaturated fatty acids have been shown to regulate mitochondrial properties and functions including calcium homeostasis, mitochondrial gene expression, respiratory function, reactive oxygen species production, mitochondrial apoptosis and biogenesis (Flachs et al., 2005; Rohrbach, 2009; Lanza et al., 2013; Afshordel et al., 2014; de Oliveira et al., 2017; Isesele and Mazurak, 2021). Still under debate, these findings reveal a link between PUFAs and mitochondrial physiology, which is supported by an increase in the abundance of mitochondria within white matter microglia without affecting other organelles.

In the last decade, the ground-breaking field of immunometabolism has grown exponentially, providing new understanding of how immune cells control their states and functional properties by adapting intracellular metabolic pathways to their needs in energy (O’Neill et al., 2016; Borst et al., 2019; Paolicelli and Angiari, 2019). Emerging evidence suggests that any phenotypic shift of microglia relies onto a metabolic reprogramming of the cells (Nadjar, 2018; Borst et al., 2019; Paolicelli and Angiari, 2019). Our data showed a decreased number of myelin debris within microglia, suggestive of altered phagocytic activity, associated with an increase in the number of mitochondria. To date, there is limited data on the metabolic shift associated to microglial phagocytosis. *In vitro* evidence, however, suggest that the modulation of phagocytosis relies on mitochondrial oxidative phosphorylation (Gimeno-Bayón et al., 2014; Orihuela et al., 2016). Hence, the increase in mitochondrial number observed in n-3 deficient mice might sustain the modulation of their phagocytic activity.

In conclusion, we uncovered a novel effect of maternal n-3 PUFA dietary deficiency on white matter microglia in the developing brain. Further studies are needed to (1) understand the mechanism by which n-3 PUFA affect white matter microglia during neurodevelopment, and (2) test for the causal link between white matter microglia alterations and the myelination deficits that are observed in n-3 deficient mice.

## Data Availability Statement

The original contributions presented in the study are included in the article/supplementary material, further inquiries can be directed to the corresponding author/s.

## Ethics Statement

The animal study was reviewed and approved by Direction Départementale de la Protection des Animaux, approval ID: A33-063-920, #15517.

## Author Contributions

FD, KP, AS, M-KS-P, AG, and J-CD performed all the experiments. FD, KP, and AS performed the mice perfusion. AG and J-CD supervised part of the experiments. FD and KP performed the immunochemistry staining and tissue preparation for the electron microscopy. FD, KP, and M-KS-P cut the sample for electron microscopy. FD performed the electron microscopy imaging and analyzed the acquired images with the help of M-KS-P. SL provided part of the financial support and facilities necessary for the project. AN and M-ET led the entire project. AN, M-ET, and FD wrote the manuscript. All authors proofread the manuscript.

## Conflict of Interest

The authors declare that the research was conducted in the absence of any commercial or financial relationships that could be construed as a potential conflict of interest.

## Publisher’s Note

All claims expressed in this article are solely those of the authors and do not necessarily represent those of their affiliated organizations, or those of the publisher, the editors and the reviewers. Any product that may be evaluated in this article, or claim that may be made by its manufacturer, is not guaranteed or endorsed by the publisher.
